# The Thienopyrimidinone Gamhépathiopine Targets the Q_O_ Site of Plasmodium falciparum Cytochrome *b*

**DOI:** 10.1021/acsinfecdis.5c00259

**Published:** 2025-06-03

**Authors:** Natalie Wiedemar, Rachel Milne, Sandra Carvalho, Stephen Patterson, Mike Bodkin, Nicolas Masurier, Vincent Lisowski, Nicolas Primas, Pierre Verhaeghe, Graeme M. Sloan, Susan Wyllie

**Affiliations:** a Wellcome Centre for Anti-Infectives Research, Division of Biological Chemistry and Drug Discovery, School of Life Sciences, 98264 University of Dundee, Dow Street, Dundee DD1 5EH, United Kingdom; b Drug Discovery Unit, Wellcome Centre for Anti-Infectives Research, Division of Biological Chemistry and Drug Discovery, School of Life Sciences, University of Dundee, Dow Street, Dundee DD1 5EH, United Kingdom; c 56828 Institut des Biomolécules Max Mousseron, UMR 5247, CNRS, Université de Montpellier, ENSCM, UFR des Sciences Pharmaceutiques et Biologiques, 34093 Montpellier, France; d Department of Pharmacy, Lapeyronie Hospital, CHU Montpellier, 191 Av. du Doyen Gaston Giraud, 34295 Montpellier, France; e AP-HM, Service Central de la Qualité et de l’Information Pharmaceutiques, Hôpital Conception, 13005 Marseille, France; f Aix Marseille Univ, CNRS, ICR UMR 7273, Equipe Pharmaco-Chimie Radicalaire, Faculté de Pharmacie, 13385 Marseille, France; g LCC-CNRS Université de Toulouse, CNRS, UPS, 31062 Toulouse, France; h Université de Grenoble Alpes, CNRS, DPM UMR 5063, 38041 Grenoble, France

**Keywords:** malaria, thienopyrimidinone, cytochrome *b*, Q_o_ active site, drug discovery, mode of action

## Abstract

Chemotherapy remains a key component of the arsenal of tools to fight malaria. Specifically, new drugs with diverse mechanism(s) of action are required to combat existing drug resistance. Here, we describe comprehensive studies to determine the molecular target(s) of gamhépathiopine, a thienopyrimidinone showing promise for the treatment of malaria. *In vitro* evolution of gamhépathiopine resistance and whole genome analyses identified mutations within the Q_O_ site of Plasmodium falciparum cytochrome *b*, part of complex III of the electron transport chain. Subsequent biochemical assays demonstrated that gamhépathiopine directly inhibits complex III activity. Furthermore, exogenous expression of Saccharomyces cerevisiae dihydroorotate dehydrogenase, known to render the electron transport chain dispensable in *Plasmodium*, results in complete abrogation of gamhépathiopine activity. Cross-resistance profiling and docking studies indicate that gamhépathiopine occupies a similar, but not identical, binding pose to the established Q_O_-targeting antimalarial atovaquone. The implications of these findings for the future development of gamhépathiopine are discussed.

## Graphical abstract

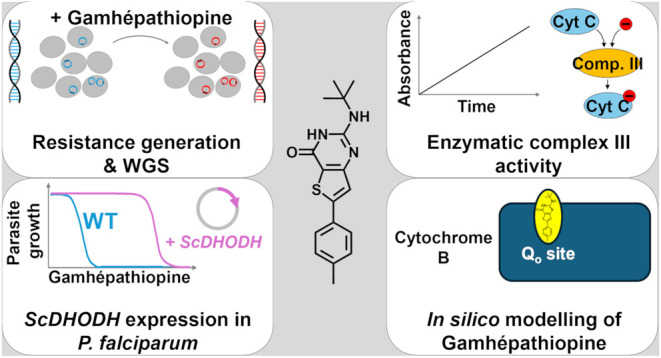

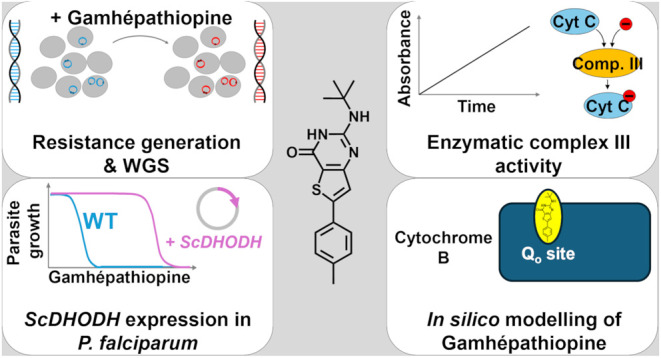

Malaria is caused by infection with *Plasmodium spp*. with Plasmodium falciparum and Plasmodium vivax responsible for the majority of severe cases. Transmitted through the bite of infected female *Anopheles* mosquitoes, malaria remains a major global killer with an estimated 249 million cases and >600,000 deaths annually.^1^ Current treatment regimens are heavily reliant upon various artemisinin-based combination therapies (ACT). However, the effectiveness of ACT is now threatened due to the emergence of artemisinin clinical resistance. Artemisinin-resistant P. vivax isolates were first identified over 20 years ago in Southeast Asia.^1,2^ More recently, artemisinin-resistant P. falciparum isolates have emerged in Sub-Saharan Africa and are of particular concern since the vast majority of the global disease burden occur in this region. Two malaria vaccines, RTS,S/AS01 and R21/Matrix-M, have recently been approved and are currently being administered in Sub-Saharan Africa.^3^ Both vaccines are safe, effective in children and prevent ∼75% of malaria episodes in areas of high season transmission. While both vaccines will likely have huge public health benefits, it should be noted that the protection provided is of relatively short duration, thus regular and sustained vaccination programs will be required. With this in mind, it is clear that effective chemotherapeutics are likely to remain a vital component of future malaria control and eradication strategies. Developing new drugs capable of treating artemisinin-refractory infections is of the highest priority. In addition, drugs that prevent human-to-mosquito transmission through targeting gametocytes as well as those targeting hepatic stages of the parasite, including dormant P. vivax hypnozoites, are urgently required.

In search of suitable chemical start points for antimalarial drug discovery, Cohen and colleagues screened compounds from the CNRS French Library against P. falciparum intraerythrocytic parasites.^4^ Screening identified a thieno­[3,2-*d*]­pyrimidin-4­(3*H*)-one scaffold demonstrating antimalarial activity in the nM range. Subsequent hit expansion and optimization led to the development of a lead compound, gamhépathiopine (2-*tert*-butylaminothieno­[3,2-*d*]­pyrimidin-4­(3*H*)-one, Figure 1A).^5^ Importantly, this compound retained activity against artemisinin- and multidrug-resistant P. falciparum cell lines, was confirmed as gametocidal and also active against liver-stage Plasmodium yoelii, P. falciparum, and Plasmodium cynomolgi *in vitro*. Unfortunately, gamhépathiopine demonstrated only partial activity in mouse models of infection, with this lack of efficacy attributed to rapid *in vivo* metabolism as evidenced by an 11 min half-life in mouse microsomal assays.^5^ Subsequent development of the series focused on increasing metabolic stability and improving permeability.^6–10^ While thienopyrimidinone derivatives with improved half-lives (>70 min) were identified,^8^ it became clear that considerable modification of the series would be required to fulfill the malaria chemoprevention target product profile, which requires pharmacological duration of protection of at least a month.

**Figure 1:**
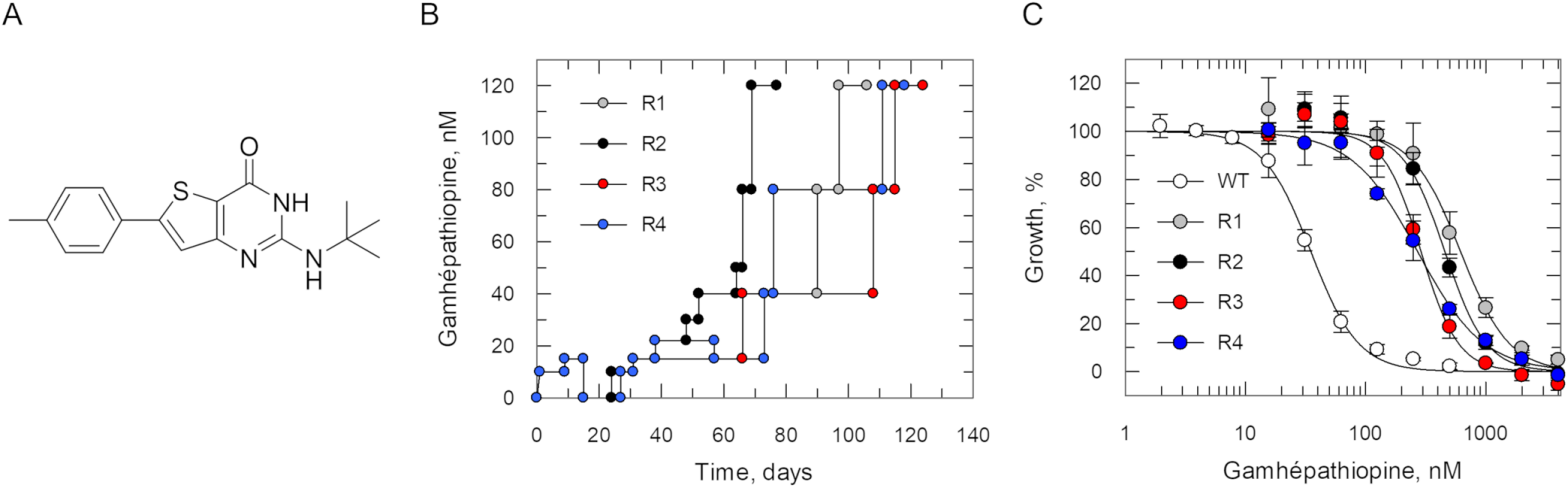
*In vitro* evolution of resistance to gamhépathiopine in P. falciparum. (A) Chemical structure of gamhépathiopine. (B) Schematic representation of the generation of gamhépathiopine-resistant cell lines in P. falciparum (Dd2). Each passage of cells in culture (circles, lines 1–4) is indicated with cell lines 1–4 indicated in gray, black, red and blue, respectively. (C) Dose–response EC_50_ values for gamhépathiopine were determined for WT (white) and cloned resistant cell lines 1–4 (gray, black, red and blue, respectively). These representative curves are the nonlinear fits of data using a two-parameter EC_50_ equation provided by GraFit. An EC_50_ value of 35 ± 1 nM was determined for gamhépathiopine against the parental WT. EC_50_ values for resistant clones gam-R1–4 were 616 ± 47, 456 ± 29, 289 ± 14 and 270 ± 12 nM, respectively. These EC_50_ values represent one biological replicate, composed of two technical replicates. Collated data sets reporting the weighted mean ± SD of multiple biological replicates are summarized in Table 1.

Lack of information regarding the mechanism(s) of action or molecular target(s) of phenotypically active compounds can prove a significant barrier to optimization. Here, we utilized orthogonal genetic, molecular, and biochemical studies to identify the primary target of gamhépathiopine in P. falciparum. Our comprehensive studies indicate that this compound interacts with and inhibits cytochrome *b*, part of the cytochrome *bc1* complex of the electron transport chain. Gamhépathiopine resistance-conferring mutations cluster in the Q_o_ active site of cytochrome *b* enabling the binding site of this thienopyrimidinone to be predicted. The implications of this mechanism of action for the future development of this series and ways this information can be used to guide the development of derivatives with improved pharmacokinetic properties are discussed.

## Results and Discussion

### Resistance Generation Followed by Whole Genome Sequencing

As a first step toward target identification, cell lines resistant to gamhépathiopine were selected through *in vitro* evolution. Starting at 10 nM, four drug-sensitive, clonal P. falciparum Dd2 cultures were exposed to stepwise increasing concentrations of gamhépathiopine until they were capable of growth in 120 nM (∼10× the established EC_50_ value). Selection of this level of resistance was achieved in 60–120 days (Figure 1B). The four independently generated resistant cell lines were subsequently cloned by limiting dilution, and the susceptibility of the resulting clones to gamhépathiopine was assessed. Resistant clones (gam-R1–4) were between 12- and 30-fold less sensitive to the compound than the wild-type (WT) parental clone (Table 1 and Figure 1C). One clone was selected at random (gam-R2) and maintained in culture for 6 weeks in the absence of compound selection. Following this period of compound-free culture, reanalysis confirmed that gam-R2 remained resistant to gamhépathiopine (24-fold) indicating that the resistance phenotype is relatively stable.

**Table 1:** Collated EC_50_ Values for WT, Resistant and Transgenic Cell Lines

	gamhépathiopine			atovaquone		
cell line	^a^EC_50_ values, nM	fold shift (relative to WT)	biological replicates	^a^EC_50_ values, nM	fold shift (relative to WT)	biological replicates
Dd2	16 ± 0.7		23	0.4 ± 0.02		15
Gam-R1	461 ± 37	28	5	2 ± 0.2	4.5	3
Gam-R2	496 ± 34	30	4	10 ± 0.6	22	3
Gam-R3	198 ± 13	12	5	1 ± 0.08	2.5	3
Gam-R4	301 ± 18	18	5	0.8 ± 0.07	2	3
NF54-AttB	16 ± 1		8	0.6 ± 0.06		8
*Sc*DHODH-C1	8913 ± 920	507	4	43,906 ± 3267	70,040	4
ScDHODH-C2	7943 ± 414	580	4	41,677 ± 3665	66,484	4

a EC_50_ values represent the weighted mean ± SD of ≥3 biological replicates with each biological replicate comprised of ≥2 technical replicates.

Genomic DNA recovered from the four compound-resistant clones was analyzed by whole genome sequencing (WGS). In comparison to the gamhépathiopine-sensitive parent clone, 10 nonsynonymous sequence variants were identified (Table 2). Of particular note, all 4 resistant clones harbored mutations in the gene encoding cytochrome *b* (PF3D7_MIT02300), a key component of complex III of the *Plasmodium* electron transport chain. Cytochrome *b* contains 2 discrete reaction sites involved in the Q cycle: a ubiquinone reduction center (Q_i_ site) and a ubiquinol oxidation center (Q_o_ site). Four of the five cytochrome *b* mutations (G131S, N250S, Q257E and L265I) identified in gamhépathiopine-resistant parasites mapped to the Q_o_ center of the enzyme. The remaining mutation (V284G) was located in the sixth transmembrane domain, adjacent to the Q_0_ site.^11^

**Table 2:** SNPs and INDELS Identified in Gamhépathiopine-Resistant Clones

gene ID	function	R1	R2	R3	R4
PF3D7_0301600	*plasmodium* exported protein (hyp1), unknown function				R625S
PF3D7_0710000	conserved *plasmodium* protein, unknown function				K2554R
PF3D7_0914200	phospholipid or glycerol acyltransferase, putative				F264Y
PF3D7_1216900	DNA-binding chaperone, putative		N250dup		
PF3D7_1462800	glyceraldehyde-3-phosphate dehydrogenase	A216S			
PF3D7_MIT02300	cytochrome *b*	G131S, L265I	V284G	N250S	G131S, Q257E

### Cross-Resistance Profiling

Atovaquone is administered in combination with proguanil under the trade name Malarone for the treatment and prevention of malaria. This naphthoquinone specifically targets the Q_o_ site of *Plasmodium* cytochrome *b*,^12–14^ acting as a competitive inhibitor of ubiquinol. Resistance to atovaquone is commonly driven by mutations at the Q_o_ active site.^13,15,16^ Of the identified amino acid substitutions in our gamhépathiopine-resistant clones, V284 is the only residue previously reported as mutated in the context of atovaquone resistance (Plasmodium berghei, V284F).^17^ However, all of our clones demonstrated some degree of cross-resistance to atovaquone ranging from 2–22-fold (Table 1), with gam-R2 bearing the V284G mutation unsurprisingly the most cross-resistant. Collectively, these data provide further evidence that gamhépathiopine is a Q_o_ site inhibitor and indicates that this compound may exploit an overlapping but not identical binding site as atovaquone.

### Inhibition of Complex III Activity

Complex III of the electron transport chain is comprised of cytochrome *b*, cytochrome *c1*, and the Rieske iron–sulfur protein. This complex, embedded in the inner mitochondrial membrane, facilitates the transfer of electrons from ubiquinol to cytochrome *c* through a process known as the Q cycle.^18,19^ The cycle is composed of two half-cycles. In each half-cycle, ubiquinol is oxidized to ubiquinone via transfer of two electrons at the Q_o_ site of cytochrome *b*. One electron reduces cytochrome c, while the second is transferred to the Q_i_ site of cytochrome *b*, where it reduces ubiquinone to ubiquinol. The complete cycle results in the concomitant efflux of 4 protons into the mitochondrial intermembrane space thereby maintaining the proton gradient essential for ATP production.

To directly monitor the effect of gamhépathiopine on complex III activity, lysates of blood-stage P. falciparum enriched for membranes (including mitochondrial membranes) were prepared. Using decylubiquinol as a pseudosubstrate, the activity of complex III in the presence and absence of test compounds was determined by monitoring the reduction of cytochrome *c* at 550 nm. The assay was validated using atovaquone as a positive control and DDD01510706, an unrelated inhibitor of lysyl-tRNA synthetase,^20^ as a negative control. Activities were benchmarked against lysates incubated with compound diluent (1% DMSO). As expected, atovaquone (100 nM) almost completely ablated complex III activity while DDD01510706 (20 μM) had little or no effect. Incubation with gamhépathiopine led to a marked, dose-dependent reduction in complex III activity. Indeed, at the highest concentration of gamhépathiopine tested (20 μM), activity was reduced by >93% (Figure 2).

**Figure 2:**
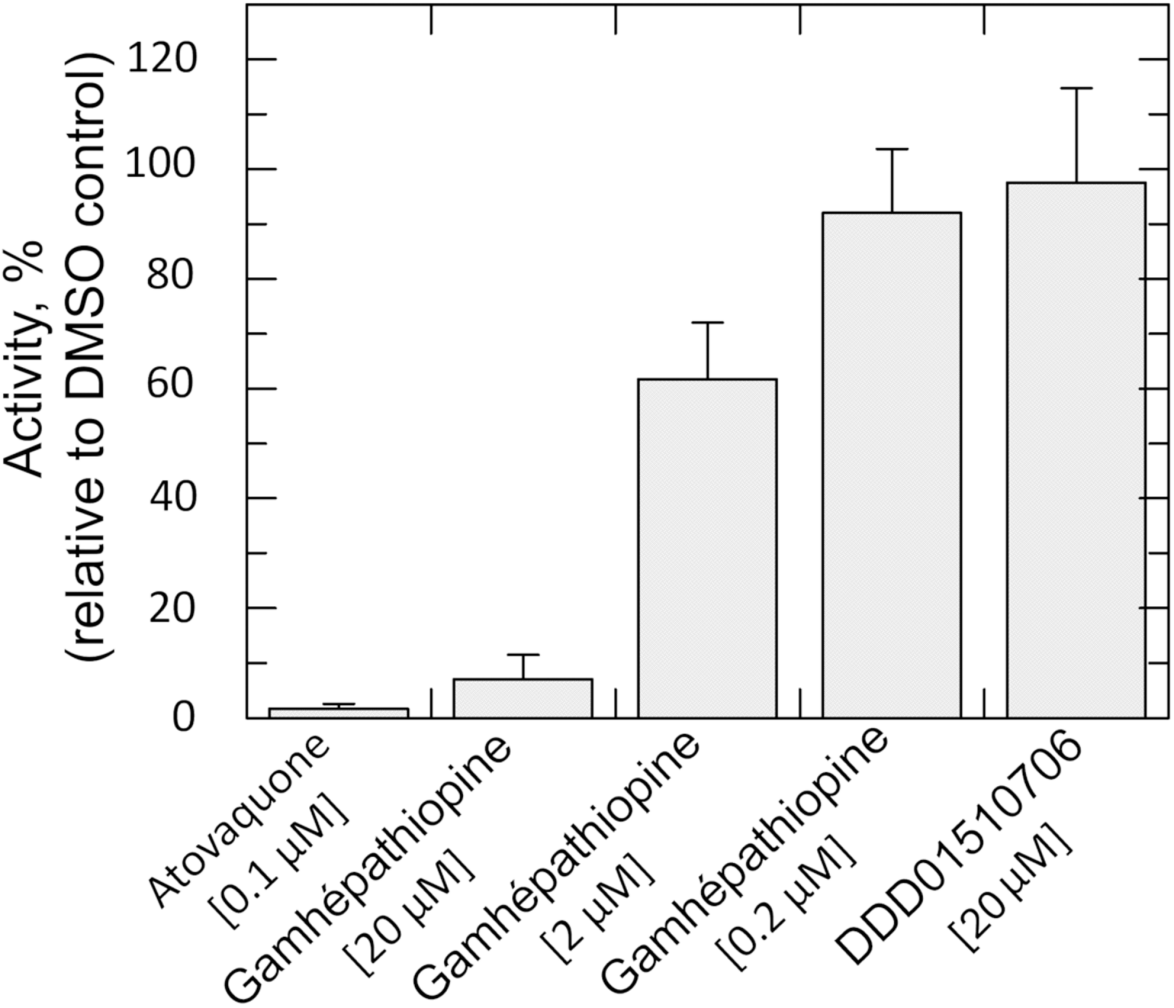
Inhibition of P. falciparum complex III activity. Complex III activity was determined in the presence of different concentrations of gamhépathiopine, using atovaquone as positive inhibition control and compound DDD01510706 as a negative control. Data are derived from two independent biological replicates each comprised of three technical replicates.

Previous studies with gamhépathiopine sought to assess the impact of compound treatment (1× EC_50_, 20 min) on the P. falciparum mitochondrial membrane potential.^4^ We hypothesize that the inhibitor concentration and duration of incubation in these preliminary studies was insufficient to elicit an effect at the cellular level. Undoubtedly, prolonged incubation with higher concentrations of this compound will result in the ultimate collapse of membrane potential.

### Expression of Saccharomyces cerevisiae DHODH in P. falciparum

Intraerythrocytic *Plasmodium* parasites heavily rely on glycolysis for ATP-production;^21^ thus, the predominant function of the mitochondrial electron transport chain is to regenerate ubiquinone to serve as an electron acceptor for dihydroorotate dehydrogenase (DHODH) in the *de novo* pyrimidine biosynthesis pathway.^22^ Previous studies have demonstrated that cytosolic S. cerevisiae DHODH (*Sc*DHODH) can compensate for loss of P. falciparum DHODH function, with the resulting parasites using fumarate as an electron acceptor. This means that in asexual blood-stage parasites expressing *Sc*DHODH the electron transport is effectively dispensable^22^ and inhibitors targeting this pathway ineffective. As a complementary approach to confirm gamhépathiopine-mediated inhibition of the electron transport chain and to assess the impact of potential secondary targets, transgenic blood-stage P. falciparum expressing *Sc*DHODH were generated (Figure 3A). Briefly, the NF54-AttB cell line was cotransfected with the pINT plasmid, harboring a gene encoding the mycobacterial Bxb1 integrase, and the AttP-plasmid^23^ harboring *ScDHODH* and *BSD*. Expression of the Bxb1 integrase catalyzes the accurate integration of transgenes into the *cg6*-*attB* locus of NF54-AttB parasites. Transfected parasites were selected with blasticidin and cloned by limiting dilution. Two clones were randomly selected for further characterization. Subsequent PCR and Sanger sequencing confirmed the correct genomic integration of *ScDHODH*. In addition, quantitative RT-PCR detected *ScDHODH* transcripts indicating that the transgene was successfully transcribed in these transgenic clones (Figure S1). As expected, *Sc*DHODH-expressing clones were significantly less susceptible (>65000-fold, Figure 3B and Table 1) to the established cytochrome *b* Q_o_ inhibitor atovaquone than wild-type parasites. Reassuringly, these transgenic clones also demonstrated a marked drop in sensitivity to gamhépathiopine (>500-fold, Figure 3C and Table 1). Collectively, our data confirms that gamhépathiopine is a selective inhibitor of P. falciparum cytochrome *b* and likely interacts specifically with the Q_o_ active site of this enzyme.

**Figure 3:**
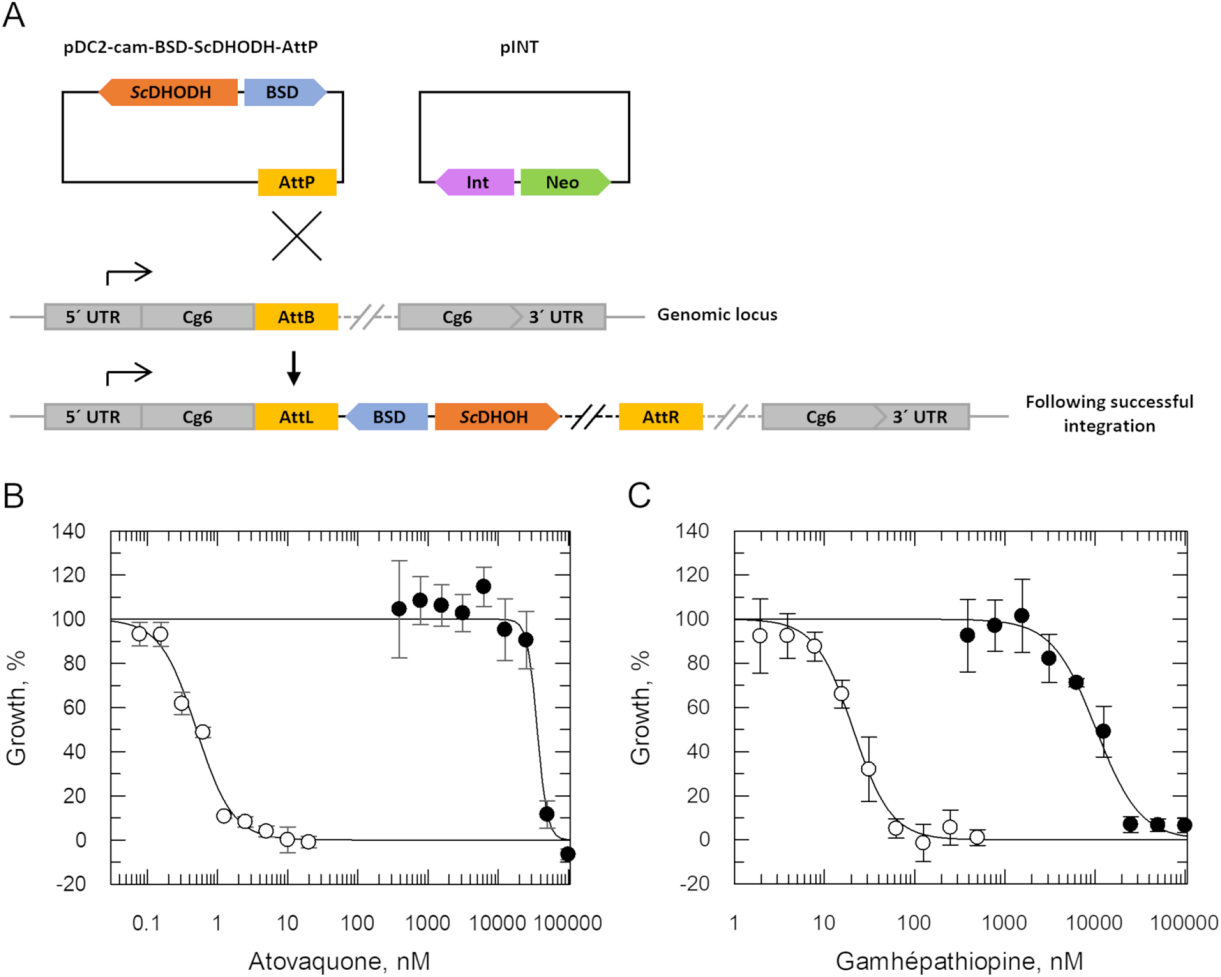
Expression of S. cerevisiae DHODH in P. falciparum. (A) Schematic representation of the cloning strategy to introduce a *ScDHODH* expression cassette into the pre-edited *cg6* locus of the P. falciparum NF54 cell line.^23^ A plasmid containing *ScDHODH*, blasticidin-S-deaminase (BSD) and an AttP site was cotransfected with pINT containing a viral integrase (Int) and the neomycin resistance gene (Neo). (C, D) Dose–response curves of the NF54-AttB parental cell line and a *Sc*DHODH-expressing clone (C1) with atovaquone (C) and gamhépathiopine (D), respectively. For atovaquone, the EC_50_ of the parental NF54 line was 0.5 ± 0.04 nM, and 40,632 ± 3829 nM for the *Sc*DHODH expressing clone. For gamhépathiopine, the parental NF54 line had an EC_50_ value of 20.8 ± 1.3 nM, and 10,304 ± 1052 nM for the *Sc*DHODH-expressing clone. Data show one representative assay consisting of three technical replicates. Data of multiple independent assays are shown in Table 1.

### Exploring Gamhépathiopine Binding Site within the Q_o_ Pocket of Cytochrome *b*

With the aim of defining the binding site of gamhépathiopine and understanding the role of Q_o_ mutations in gamhépathiopine resistance, a predicted structural model of the P. falciparum cytochrome *b* was recovered from the AlphaFold database and further refined using Homo sapiens and Pseudomonas aeruginosa orthologue models. The P. falciparum model was scoped to identify potential binding sites using Schrodinger’s SiteMap and once identified, gamhépathiopine and the established Q_o_ inhibitor atovaquone were docked into these sites. In the best docking poses, atovaquone and gamhépathiopine exploit overlapping binding sites within the Q_o_ pocket (Figure 4). In keeping with previous structural studies, the hydroxynaphthalenedione moiety of atovaquone orientated toward Met133, with the cyclohexyl and 4-chloro-phenyl moieties occupying a hydrophobic pocket, likely forming π–π contacts with Phe267 and hydrophobic contacts with additional side chains. Gamhépathiopine is predicted to bind in the same pocket, with the tolyl group of the compound pointing toward Met133. The thiophene portion of the bicyclic occupies a similar position to the atovaquone cyclohexyl, with the *tert*-butylamine extending out of the primary pocket toward hydrophobic contacts with Val120, Phe123 and Ile141.

**Figure 4:**
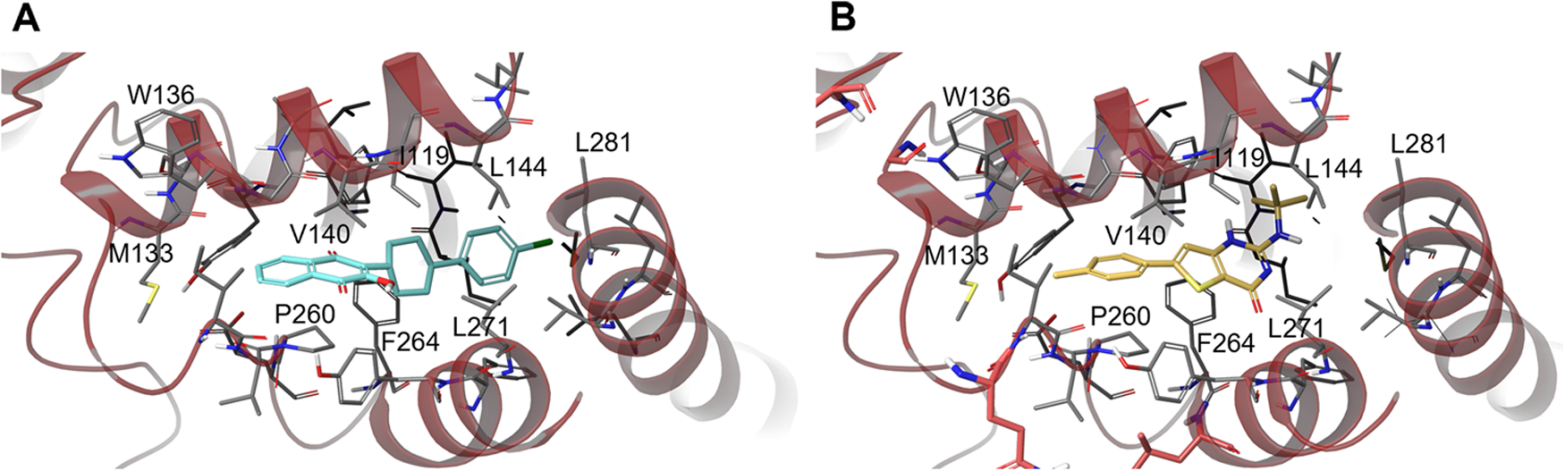
Docked poses for atovaquone (A, teal) and gamhépathiopine (B, yellow) in the Q_o_ pocket of P. falciparum cytochrome *b*.

Three of the residues mutated in our gamhépathiopine-resistant cell lines (G131, N250 and Q257) are located in close proximity to, but not directly inside, the binding pocket (Figure 5). The location of these residues indicate that these mutations are likely to constrain gamhépathiopine binding in an indirect rather than direct manner, in keeping with the comparatively modest levels of resistance they confer. The remaining two mutated residues (L265 and V284) are within the predicted binding sites of both gamhépathiopine and atovaquone. However, the leucine side chain points down and away from the ligand rather than pointing inward (Figure 5). We hypothesize that the L265I mutation, and the increased steric bulk in the isoleucine side chain, significantly impacts the shape and volume of the binding pocket, with nearby residues moving to accommodate the mutation. In contrast, the V284 side chain points inward toward gamhépathiopine. In this case, the V284G mutation reduces the steric bulk. While no formal interactions with the ligand are disrupted based on our *in silico* model, this change is likely to impact the surrounding residues since the space previously occupied by the valine side chain will be exploited by neighboring residues. In both cases, these resistance-conferring mutations likely induce significant binding site rearrangements that likely diminish gamhépathiopine binding thus conferring resistance. The *tert*-butyl group of gamhépathiopine may cause the ligand to be more susceptible to disruption of binding due to the increased steric bulk in comparison to atovaquone. This is consistent with the fact that the described mutations impact gamhépathiopine susceptibility more significantly than atovaquone (Table 1).

**Figure 5:**
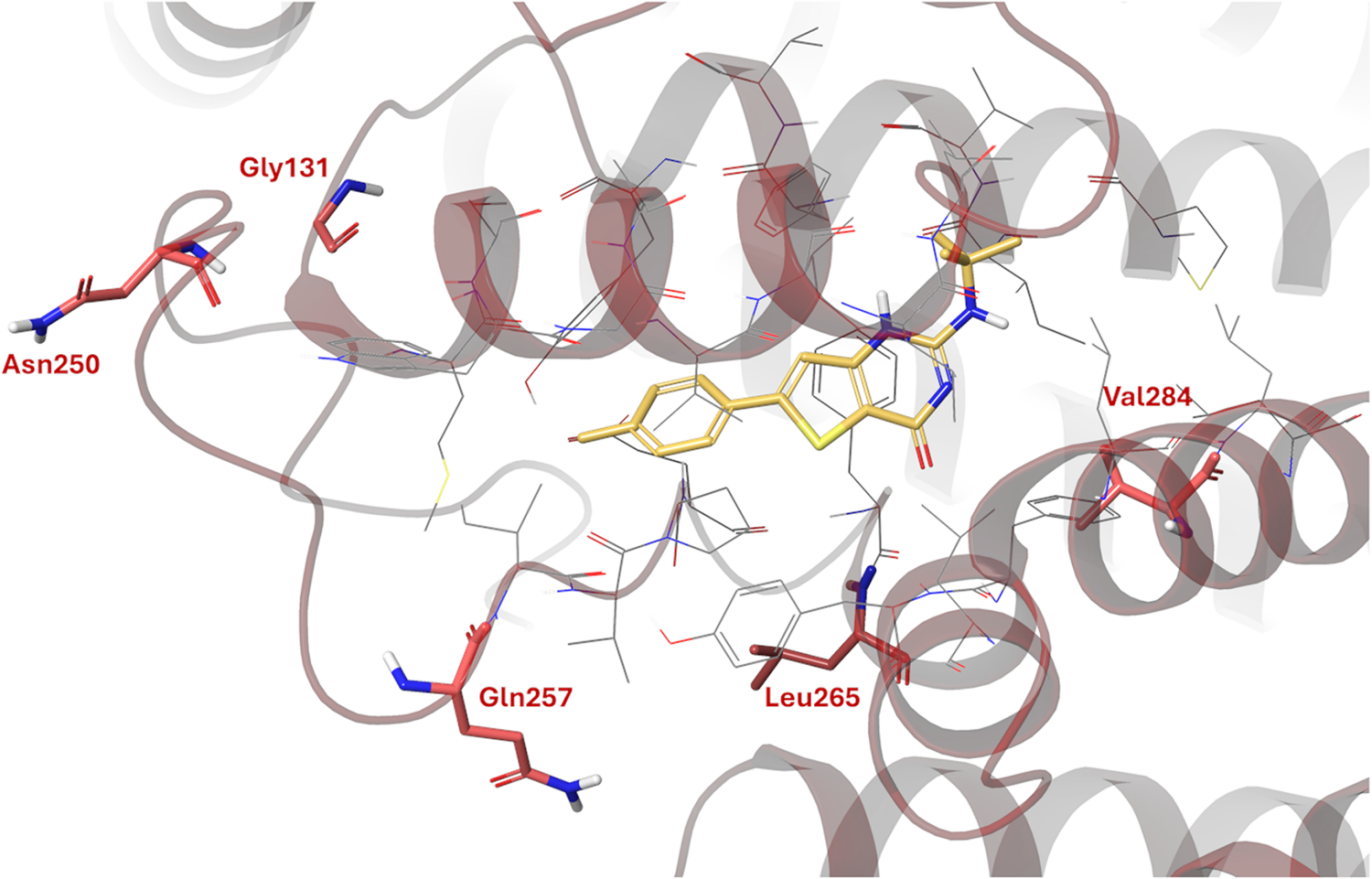
Location of resistance-conferring mutations in P. falciparum cytochrome *b* in relation to the proposed binding site of gamhépathiopine. Mutated residues are highlighted in red and gamhépathiopine in yellow.

## Conclusions

Knowledge of a compound’s molecular target is highly beneficial for decision-making during the drug development process. Indeed, knowledge of the molecular target is often crucial in developing strategies to overcome issues such as improving potency and selectivity against the pathogen of interest and reducing toxicity against the mammalian host. Compound series with favorable modes of action can be prioritized and those targeting less favorable targets efficiently deprioritized. Specifically, identification of cytochrome *b* as the molecular target of gamhépathiopine will enable subsequent development of this thienopyrimidinone series to be structure-guided and selectivity over the human cytochrome *b* homologue to be maximized.

Cytochrome *b* is genetically essential in asexual blood-stage *Plasmodium* spp. and is a clinically validated malaria drug target, with the Q_o_ site inhibitor atovaquone in the clinic. As with all antimalarials, atovaquone is prescribed as part of a combination therapy, alongside proguanil (Malarone), to reduce the risk of emerging resistance. However, in patients treated with Malarone, clinical resistance has arisen, mediated almost exclusively by a single point mutation at position Y268 of cytochrome *b*.^23^ Studies by Balta and colleagues have demonstrated that parasites bearing this mutation suffer a severe-to-lethal fitness cost and cannot be transmitted by mosquitoes perhaps limiting the transmissibility of atovaquone resistance in the field.^24^ None of the gamhépathiopine-resistant clones generated in the course of our studies bear the Y268 mutation albeit the neighboring residue L265 was mutated and associated with significant resistance to gamhépathiopine and atovaquone. Further studies will be required to assess the fitness profile of parasites bearing this and other gamhépathiopine resistance-conferring mutations. It should be noted that the current target product profile for malaria states that future antimalarials must be capable of treating existing drug-resistant clinical isolates. Thus, development of this thienopyrimidinone series should focus on further differentiating the binding pose from that of atovaquone in order to minimize the potential for cross-resistance.

Over the past few years, multiple inhibitors of *Plasmodium* cytochrome *b* have been identified through phenotypic screening and target deconvolution with the vast majority bearing quinolone, quinoline, or naphthoquinone cores.^25–28^ To our knowledge, this represents the first time that an inhibitor bearing a thienopyrimidine core has been found to target this enzyme. These studies expand our understanding of chemotypes capable of inhibiting this vitally important antimalarial drug target.

## Experimental Section

### Ethics Statement

Parasites were cultured in fresh human erythrocytes obtained with ethical approval from anonymous healthy donors, with informed written consent as part of the recruitment process, from the Scottish National Blood Transfusion Service (SNBTS). The use of erythrocytes was approved by the University of Dundee Schools of Medicine and Life Sciences Research Ethics Committee (Approval reference: 21/39).

### Cell Lines and Culture

P. falciparum asexual blood stage parasites (Dd2 and NF54) were cultivated in type A+ red blood cells (RBC) were grown at 5% hematocrit and kept between 0.5–5% parasitemia, in complete malaria media (CMM: RPMI 1640 containing 25 mM HEPES and 2 mM l-glutamine [Gibco] supplemented with 0.5% Albumax II [Gibco], 12 mM sodium bicarbonate, 11 mM glucose, 0.2 mM hypoxanthine, and 20 mg/L gentamicin, pH 7.3). Parasites were cultivated in a humidified atmosphere at 1% O_2_ and 3% CO_2_ in a balance of N_2_ at a temperature of 37 °C.

Synchronization of parasites for transfection and mRNA isolation was achieved by sorbitol treatment. Briefly, the cultures were centrifuged (1500*g*, 5 min, RT), and the RBC pellet eluted in 9 volumes of 5% sorbitol and incubated for 5 min on ice to lyse RBCs infected with trophozoite and schizont stage parasites. Cells were washed once with CMM and returned to culture.

### Drug Sensitivity Assays

Serial drug dilutions in CMM were set up in 96-well tissue culture plates. Parasites and RBCs were added to a final concentration of 2.5% hematocrit and 0.6% parasitaemia in a total volume of 100 μL per well. Mefloquine, 10 μM, served as 100% inhibition control. Plates were incubated for 72 h. Subsequently, 50 μL cell lysis buffer (20 mM Tris-HCl, 5 mM EDTA, 0.16% saponin w/v, 1.6% TX100 v/v, pH 7.9) with 3× SYBRGreen [Thermo Fisher] reagent was added to the plates. The plates were incubated in the dark at RT for 3–4 h, and fluorescence (excitation 485 nm and emission 528 nm) was quantified using a Tecan Infinite Pro 200 microplate reader. Dose–response curves and effective concentrations inhibiting parasite growth by 50% (EC_50_) were calculated with a two-parameter equation in GraFit version 7.0 (Erithacus Software) shown below

$$y=\frac{100}{1+{\left(\frac{\left[I\right]}{{\mathrm{EC}}_{50}}\right)}^{m}}$$

­[I] represents the inhibitor concentration, and *m* is the slope factor. Experiments were performed in, at least, three independent biological replicates, and the data are presented as the weighted mean ± standard deviation.

### Parasite Cloning

Based on the assumption that 1 mL of packed hematocrit contains 10^10^ RBCs, the parasite cultures were diluted to 2% hematocrit and 2.5 parasites/mL. Two hundred microliters of the parasite dilution were added to each well of a 96-well plate and incubated. Every 7 days, 100 μL of media was replaced with medium containing fresh RBCs. After 17–19 days, 40 μL were transferred to a new plate containing 20 μL of cell lysis buffer with SYBR-Green reagent (see above). Plates were incubated and fluorescence measured as described above. Positive wells were transferred to fresh culture medium at 5% hematocrit.

### Resistance Generation

A Dd2 clone was exposed to stepwise increasing concentrations of gamhépathiopine in four independent replicates. Starting at 10 nM (slightly below the initially determined EC_50_ of 12 nM), the drug concentration was increased as soon as parasites showed growth comparable to a no-drug control culture. The drug concentration was decreased when growth stagnated. Once parasites showed stable growth at 120 nM (10× the initial EC_50_), the lines were cloned, the drug sensitivity determined, and the DNA isolated and sent for whole genome sequencing.

### DNA Isolation and Whole Genome Sequencing

To isolate the genomic DNA, 100 mL parasite culture were centrifuged (1800*g*, 5 min, RT), the supernatant removed, the pellet eluted in 5 volumes of 0.15% saponin [Panreac Applichem] and incubated for 5 min at RT to lyse RBCs. The samples were centrifuged (2800*g*, 8 min, RT) and washed 3× with PBS. The DNA was subsequently isolated from the pellet containing the free parasites, using a standard alkaline lysis protocol.

The genomic DNA was sequenced at the Beijing Genomics Institute on a DNBSEQ-G400. A total of 5.9–16 million 100-bp paired-end sequencing reads were generated for each sample. Sequencing reads were mapped to the P. falciparum 3D7 reference genome (version 48, https://plasmodb.org) using Bowtie2 (version 2.3.5).^29^ Sequence Alignment/Map (SAM) files were converted to binary BAM files, sorted, and indexed with SAMtools (version 1.9).^30^ Small sequence variants were called with bcftools (version 1.9), mpileup (options -d8000 -Ou -C50), and bcftools call (options -cv -f GQ), and variant calling was parallelized using GNU parallel.^31^ The functional effects of the variants were annotated with SNPeff (version 5.0)^32^ based on the 3D7 reference genome and annotation (version 48, https://plasmodb.org). All high-confidence variants that were absent in the parental Dd2 clone (genotype 0/0, Phred-scaled genotype likelihoods 0, >199, >199), present in at least one of the resistant clones, and predicted to alter the amino acid sequence (snpEff annotation “HIGH” or “MODERATE”) were inspected in the Integrative Genomic Viewer (version 2.11.9) to exclude false positives.^33^

To identify copy number variants, the RPKM values for each transcript were calculated with Artemis (version 16)^34^ and analyzed in Excel to find differences between the wild-type parental and the resistant clones.

Sequencing data were deposited in the European Nucleotide Archive (accession number PRJEB61247).

### Cloning of Overexpression Constructs and Parasite Transfection

The *ScDHODH* coding sequence was amplified from plasmid pY-gC^35^ with overhang primers (*ScDHODH*-start-*Avr*II–F/*ScDHODH*-stop-*Xho*I-R) and cloned into the pDC2-cam-bsd-attP plasmid using *Avr*II and *Xho*I restriction sites.^36^ Correct cloning of the resulting pDC2-cam-bsd-*ScDHODH*-AttP plasmid was confirmed Sanger sequencing primers PfCam-5′UTR-F and PfHsp86–3′UTR-R.

The plasmids were purified with the EndoFree Plasmid Maxi Kit (Qiagen), and 50 μg of pDC2-cam-bsd-*ScDHODH*-AttP were precipitated together with 50 μg pINT^36^ using sodium acetate/ethanol. The plasmids were resuspended in 10 μL of TE buffer, and before transfection, 100 μL P3 Primary Cell Nucleofector Solution/Supplement 1 (Lonza) and 12.5 mM ATP were added.

NF54-AttB parasites were synchronized twice, and 4 mL of culture at 2.5% hematocrit and 12% parasitaemia were used for transfection. The parasites were washed with incomplete cytomix (120 mM KCl, 0.15 mM CaCl_2_, 10 mM K_2_HPO_4_/KH_2_PO_4_, 25 mM HEPES, 2 mM EGTA, 5 mM MgCl_2_, pH 7.6) at 1000*g*, RT, 3 min. The pellet was resuspended in the DNA/P3/ATP solution, distributed into two 100 μL Nucleocuvette Vessels (Lonza), and immediately transfected in a Lonza 4D Nucleofector using program CM150. The nucleocuvettes were placed on ice for 1 min, the parasites resuspended in fresh media and transferred to a prewarmed tube with 10 mL CMM and 500 μL RBCs. Parasites were placed in the incubator for 1.5 h, centrifuged at 1000*g*, RT, with low brake, resuspended in CMM, transferred to cell culture flasks, and incubated under standard conditions (see above). The next day, the media was exchanged, and antibiotic selection added (250 μg/mL G418 [Invivogen] and 5 μg/mL blasticidin S [Invivogen]). During the first week, the culture medium (with G418 and blasticidin) was changed daily; subsequently, it was changed every 2–3 days (with blasticidin only). Fresh RBCs were added on day six and 11, and on day 14 one-third of the culture was replaced with fresh CMM/5% RBCs. Parasite growth was visible after 18 days, and parasites were cloned as described above.

### PCR and qPCR

Correct genomic integration of the *ScDHODH* expression cassette was confirmed by PCR (primers: PfAttB_P1/PfAttB_P2 for the 5′ recombination site; PfAttB_P3/ScDHODH_P4 for the 3′ recombination site; and PfAttB_P1/PfAttB_P3 spanning the whole locus).

Quantitative RT-PCR was used to confirm transcription of *ScDHODH*. Cells were synchronized twice and 5–10 mL of trophozoite/schizont stage parasite culture harvested for RNA isolation. Cultures were centrifuged and RBCs lysed with 0.15% saponin (5 min at RT). Free parasites were washes 3× with PBS, eluted in 350 μL RLT lysis buffer (Qiagen) supplemented with 1% β-mercaptoethanol, and the RNA isolated with the RNeasy Mini Kit (Qiagen) including an on-column DNase digestion.

Quantitative RT-PCR was carried out with the Luna Universal One-Step RT-qPCR Kit (NEB) on an Agilent MX3005P thermocycler. RNA was prediluted 1:100 in RNase-free water; primers used for qPCR were ScDHODH-qPCR-F/ScDHODH-qPCR-R for ScDHODH and β-act-qPCR-F/β-act-qPCR-R for β-actin. The Ct-values were normalized to the housekeeping gene β-actin using the ΔΔCt method.

### Complex III Assay

A P. falciparum 3D7 culture was expanded to 1.2 L at 3.3% RBC. As soon as parasitaemia reached 7–12%, the cells were harvested by centrifugation (10 min, 1800*g*, low brake (2)), and RBCs lysed by resuspension in 5 volumes of 0.1% saponin in H-media (70 mM sucrose, 210 mM d-mannitol, 1 mM EGTA, 5 mM MgCl_2_, 5 mM KH_2_PO_4_, 4 mM HEPES, pH 7.4) and incubation for 10 min on ice. The suspension was centrifuged (2800*g*, 8 min, 4 °C, brake 5), and the pellet containing the free parasites washed 3× with H-media. The pellet was then resuspended in parasite lysis buffer (H-media supplemented with 1 tablet of Roche cOmplete EDTA-free protease inhibitor per 25 mL; and 1 mM PMSF), and parasites lysed using nitrogen cavitation (Parr; 1600 psi, 25 min on ice). The lysate was subsequently centrifuged to remove larger debris (1200*g*, 10 min, 4 °C, brake 2), and the supernatant (enriched membrane fraction) passed slowly through a CS column (Miltenyi Biotec) attached to a SuperMACS II magnet to remove hemozoin crystals. The flow-through was collected and centrifuged to precipitate the mitochondrial fraction (10,000*g*, 15 min, 4 °C), and washed twice with parasite lysis buffer. The resulting pellet was resuspended in 50 μL parasite lysis buffer, and 10 μL of the suspension were added to 480 μL of reaction mix (250 mM sucrose, 50 mM KH_2_PO_4_ (pH 7.2), 0.2 mM EDTA, 1 mM NaN_3_, 2.5 mM KCN, 100 μM equine heart cytochrome c [Sigma], 0.6 mM maltoside). The reaction mix was incubated at 37 °C with the respective drug concentrations. Baseline absorbance was measured at 550 nm (UV-2401 PC, Shimadzu), the reaction was initiated by addition of 100 μM decylubiquinone, and changes in absorbance at 550 nm were monitored to measure cytochrome c reduction.

### Molecular Modeling

Predicted structures for P. aeruginosa, P. falciparum, and Homo sapien cytochrome *b* were obtained from the AlphaFold database. Potential binding sites on the protein structures were identified using SiteMap (Schrodinger Release 2025–1, Schrodinger, LLC, New York, NY, 2025). Atovaquone and gamhépathiopine were prepared for docking using LigPrep (Schrodinger Release 2025–1, Schrodinger, LLC, New York, NY, 2025) before docking using Glide in Standard Precision (SP) mode (Schrodinger Release 2025–1, Schrodinger, LLC, New York, NY, 2025). Postdocking analysis was performed using the Molecular Mechanics/Generalized Born Surface Area (MM-GBSA) method within the Prime module of the Maestro suite (Schrodinger Release 2025–1, Schrodinger, LLC, New York, NY, 2025). The MM-GBSA calculations were carried out allowing both the ligand and protein residues within 6 Å of the ligand to minimize, using the VSGB implicit solvent model and the OPLS4 force field.

## Supplementary Material



## Notes

List of primers used in this study (Table S1), and quantification of *Sc*DHODH in wild-type and transgenic lines by qRT-PCR (Figure S1) (PDF)

## &

Institute of Parasitology, Department of Infectious Diseases and Pathobiology, Vetsuisse Faculty, University of Bern, 122 Länggassstrasse, 34295 Bern, Switzerland
