# Supplementary material for: The Thienopyrimidinone Gamhépathiopine Targets the QO Site of Plasmodium falciparum Cytochrome b
Source: ACS Infect Dis. 2025 Jun 3;11(6):1719–28. doi: 10.1021/acsinfecdis.5c00259 (PMC12172035; doi:10.1021/acsinfecdis.5c00259)
Supplement: Supplementary file 1 [file id5c00259_si_001.pdf]

SUPPORTING INFORMATION

The thienopyrimidinone gamhépathiopine targets the Q_O site of *Plasmodium falciparum* cytochrome *b*

Natalie Wiedemar[a,*,&], Rachel Milne[a], Sandra Carvalho[a], Stephen Patterson[a], Mike Bodkin[b], Nicolas Masurier[c], Vincent Lisowski[c,d], Nicolas Primas[e,f], Pierre Verhaeghe[g,h], Graeme M. Sloan[b], Susan Wyllie[a,*,]

[a]Wellcome Centre for Anti-Infectives Research, Division of Biological Chemistry and Drug Discovery, School of Life Sciences, University of Dundee, Dow Street, Dundee DD1 5EH, United Kingdom.

[b]Drug Discovery Unit, Wellcome Centre for Anti-Infectives Research, Division of Biological Chemistry and Drug Discovery, School of Life Sciences, University of Dundee, Dow Street, Dundee DD1 5EH, United Kingdom.

[c]Institut des Biomolécules Max Mousseron, UMR 5247, CNRS, Université de Montpellier, ENSCM, UFR des Sciences Pharmaceutiques et Biologiques, 34093 Montpellier, France.

[d]Department of Pharmacy, Lapeyronie Hospital, CHU Montpellier, 191 Av. du Doyen Gaston Giraud, 34295 Montpellier, France.

[e]AP-HM, Service Central de la Qualité et de l'Information Pharmaceutiques, Hôpital Conception, 13005 Marseille, France.

[f]Aix Marseille Univ, CNRS, ICR UMR 7273, Equipe Pharmaco-Chimie Radicalaire, Faculté de Pharmacie, 13385 Marseille, France.

[g]LCC-CNRS Université de Toulouse, CNRS, UPS, 31062 Toulouse, France.

[h]Université de Grenoble Alpes, CNRS, DPM UMR 5063, 38041 Grenoble, France.

[&]Current address: Institute of Parasitology, Department of Infectious Diseases and Pathobiology, Vetsuisse Faculty, University of Bern, 122 Länggassstrasse, 34295 Bern, Switzerland.

*Corresponding authors: Susan Wyllie (s.wyllie@dundee.ac.uk), Natalie Wiedemar (natalie.wiedemar@unibe.ch)

Table of contents

Table S1: List of primers used in this study.

Figure S1: Quantification of *Sc*DHODH in wild-type and transgenic lines by qRT-PCR.

Table S1: List of primers used in this study.

Primer name	Primer sequence (5´– 3´)
ScDHODH-start-AvrII-F	TAGCCTAGGATGACAGCCAGTTTAACT
ScDHODH-stop-XhoI-R	TGACTCGAGTTAAATGCTGTTCAACTT
PfCam-5'UTR-F	TGTATATTTTAAACTAGAAAAGGAA
PfHsp86-3'UTR-R	TTGGGGTGATGATAAAATGAAAGAT
PfAttB_P1	GAAAATATTATTACAAAGGGTGAGG
PfAttB_P2	GGCAGTTGGGATTCGTGAATT
PfAttB_P3	CTCTTCTACTCTTTCGAATTC
ScDHODH_P4	TGGAAGCTAAGGGTTATACA
ScDHODH-qPCR-F	GGTTTCGGGGGTATTGGAGG
ScDHODH-qPCR-R	ACCGGACTTAATTCCACCTGT
β-act-qPCR-F	AAAGAAGCAGCAGGAATCCA
β-act-qPCR-R	TGATGGTGCAAGGGTTGTAA

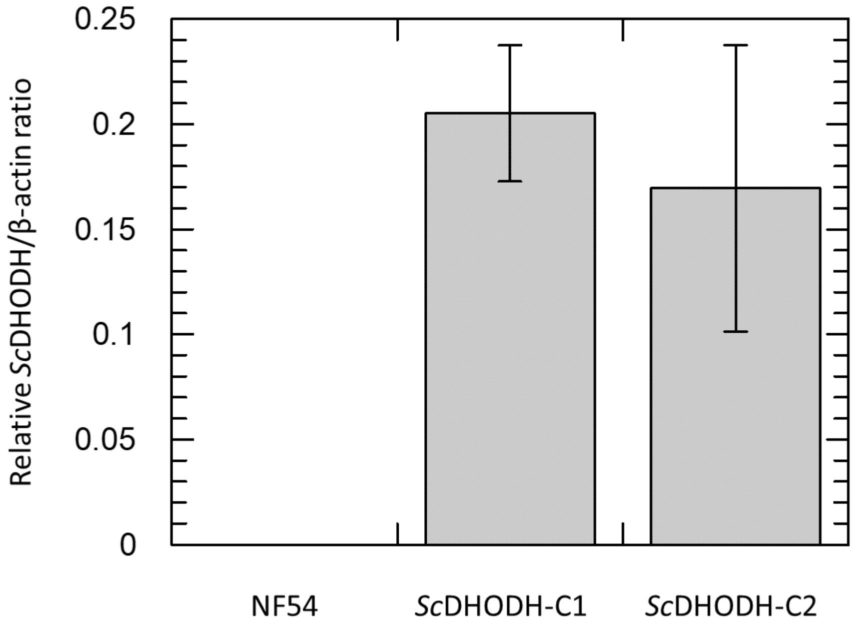

Figure S1: Quantification of *Sc*DHODH in wild-type and transgenic lines by qRT-PCR. *Sc*DHODH transcript levels relative to β-actin are shown. Details of these analyses can be found in the Materials and Methods.
